# Prognostic Implications of Pan-Cancer CMTM6 Expression and Its Relationship with the Immune Microenvironment

**DOI:** 10.3389/fonc.2020.585961

**Published:** 2021-01-20

**Authors:** Yanbin Zhao, Minghui Zhang, Haihong Pu, Shengyue Guo, Shuai Zhang, Yan Wang

**Affiliations:** Department of Medical Oncology, Harbin Medical University Cancer Hospital, Harbin, China

**Keywords:** CMTM6, pan-cancer, immune microenvironment, prognosis, The Cancer Genome Atlas

## Abstract

CKLF-like MARVEL transmembrane domain-containing 6 (CMTM6) reportedly stabilizes programmed death-ligand 1 (PD-L1) and enhances the efficacy of immunotherapy. However, correlations between CMTM6 expression and the immune microenvironment and its prognostic value remain unknown in a variety of tumors. *CMTM6* expression data were obtained from The Cancer Genome Atlas (TCGA) for 33 cancer types classified into high and low expression subgroups according to the median CMTM6 expression value. Pan-cancer analysis of CMTM6 protein expression in 20 tumor types was performed using a cohort from the Human Protein Atlas (HPA). PD-L1 protein expression data were obtained from The Cancer Proteome Atlas (TCPA) for 32 cancer types. Frequencies of CMTM6 copy number alterations and mutations were analyzed using cBioPortal. MANTIS was employed to estimate microsatellite instability in the TCGA cohort. CIBERSORT and the ESTIMATE algorithm were applied to estimate the relative fractions of infiltrating immune cell types and immune scores, respectively. Kaplan–Meier survival curve analysis was performed to assess the pan-cancer prognostic value of *CMTM6*.CMTM6 is heterogeneously expressed in diverse cancers. Further, the results revealed low *CMTM6* mutation frequencies in multiple cancers. Among them, *CMTM6* mutation frequency was the highest in uterine cancer. Additionally, CMTM6 expression was related to PD-L1 protein expression in breast invasive carcinoma, cervical squamous cell carcinoma and endocervical adenocarcinoma, cholangiocarcinoma, glioblastoma multiforme (GBM), head and neck squamous cell carcinoma, kidney renal papillary cell carcinoma, sarcoma (SARC), stomach adenocarcinoma, and uterine carcinosarcoma. Increased CMTM6 expression may be associated with increased infiltration of neutrophils in some types of cancer. Finally, pan-cancer analysis indicated that CMTM6 expression was closely related to overall survival in adrenocortical carcinoma, GBM, acute myeloid leukemia, liver hepatocellular carcinoma, mesothelioma, SARC, thymoma, and uveal melanoma. Taken together, these findings highlight that CMTM6 plays an important role in the tumor immune microenvironment, and CMTM6 has been identified to have prognostic value in some types of cancers. Thus, CMTM6 is a potential target for cancer immunotherapy and effective prognostic biomarker.

## Introduction

CKLF-like MARVEL transmembrane domain-containing family (CMTM) is a novel member of the human chemokine-like factor gene superfamily, which includes CMTM 1-8 (1). CMTM6 is a widely expressed protein that exists in clusters on human chromosome 3p23. It exhibits sequence homology with protein products of other family members and has a potential four-time membrane-penetrating structure. In recent years, the relationship between CMTM6 and tumorigenesis has attracted increasing attention. CMTM6 expression in gliomas was previously correlated with poor prognosis, and its expression was positively correlated with inhibitory T-cell expression (2). However, Joh et al. reported that CMTM6 was significantly associated with longer overall survival in non-small cell lung cancer (3). Previous studies indicate that CMTM6 plays different roles in different tumors.

Accumulating evidence indicates that monoclonal antibodies targeting programmed death-1 (PD-1) receptor and its ligand (PD-L1) have demonstrated clinical responses and survival improvement in the treatment of patients with advanced-stage cancers (4). However, anti-PD-1/PD-L1 antibodies are only efficacious in a fraction of patients with certain cancers (5). PD-L1 protein expression is a predictive biomarker that has been widely used (6). CMTM6 can be used as a key regulator of PD-L1 protein in a broad range of cancer cells. CMTM6 both stabilized PD-L1 expression and prevented its lysosome-mediated degradation. CMTM6 increases the expression level of PD-L1 protein without affecting PD-L1 transcription levels (7, 8). CMTM6 improved PD-1/PD-L1 inhibitor efficacy through modulation of PD-L1 expression and tumor-infiltrating lymphocytes (9). Additionally, CMTM6 expression was positively related to CD8+ T cell, macrophage, neutrophil, and dendtritic cell infiltration and negatively correlated with CD4+ T cell infiltration in lung squamous carcinoma (10). Therefore, fully understanding the relationship between CMTM6 and the immune microenvironment is of great importance to optimize patient benefit and guide combination approaches to treatment.

In this present study, we comprehensively analyzed the association between CMTM6 expression and the immune microenvironment and investigated its correlation with pan-cancer prognosis.

## Materials and Methods

### Pan-Cancer Analysis of CMTM6 Expression

CMTM6 mRNA expression data from 11,093 samples of normal and tumor tissues comprising 33 cancer types were downloaded from The Cancer Genome Atlas (TCGA) database (https://portal.gdc.cancer.gov/). The expression profile data were classified into high and low expression groups according to the median value of CMTM6 expression. Pan-cancer analysis of CMTM6 protein expression in 20 tumor types was performed using the Human Protein Atlas (HPA) database (https://www.proteinatlas.org/).

### Pan-Cancer Analysis of CMTM6 Copy Number Alterations and Mutations

The cBio cancer genomics portal (http://cbioportal.org) is an open-access resource capable of analyzing genomic alterations from various cancer samples (11). We used cBioPortal to identify frequencies of CMTM6 copy number alterations and mutations.

### Tumor Mutational Burden Estimates

Mutation annotation files were downloaded using the TCGAbiolinks package in R. Somatic mutation calling was performed using the MuTect2 pipeline [Genome Analysis Toolkit (GATK), Broad Institute, Cambridge, MA, USA]. The read.maf function was used to read somatic variants of each sample. The tumor mutational burden (TMB) was defined as the number of somatic variants per megabase of genome (12).

### PD-L1 Protein Expression

PD-L1 protein expression data were obtained from 6,944 tumor samples comprising 32 cancer types from The Cancer Proteome Atlas (TCPA) database (https://tcpaportal.org/tcpa/).

### Microsatellite Instability

MANTIS is a tool for identifying microsatellite instability in paired tumor-normal patient samples. MANTIS was employed to estimate MSI across 33 cancer types from the TCGA database. The average distance threshold value = 0.4 was used to distinguish microsatellite stable (MSS) tumors from those with high instability (MSI-H) (13).

### Tumor Immune Microenvironment Analysis

CIBERSORT was applied to estimate the relative fractions of 22 infiltrating immune cell types in each tumor sample using R package (14). The ESTIMATE algorithm was exploited to infer the immune scores for each sample (15).

### Statistical Analysis

Cancer patients were classified into high and low CMTM6 expression subgroups based on the median value of CMTM6 expression. The Wilcoxon test was used to evaluate expression differences between normal and tumor tissues. Overall survival (OS) was calculated using the Kaplan–Meier method, and survival curves were compared using log-rank tests. Pearson analysis was performed to evaluate the correlation between CMTM6 expression levels with checkpoint related genes. All statistical analysis was conducted using R software (version 3.6.1). P value <0.05 was considered statistically significant.

## Results

### Pan-Cancer CMTM6 Expression

**Figure 1:**
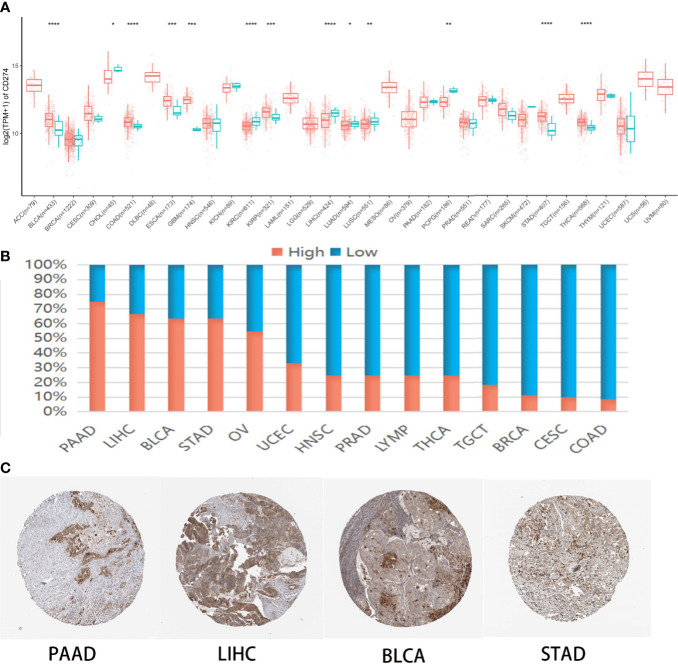
CMTM6 expression in human pan-cancer. **(A)** Differential CMTM6 mRNA expression between tumor and normal tissues in The Cancer Genome Atlas (TCGA) cohort. Red color represents cancer samples and blue color represents normal samples **(B)**. CMTM6 protein expression across 20 cancer types in Human Protein Atlas (HPA). **(C)** Representative immunohistochemical staining of CMTM6 in HPA. *p < 0.05; **p < 0.01, ***p < 0.001, ****p < 0.0001.

CMTM6 mRNA levels in >10,000 tumor and normal tissue samples were analyzed from the TCGA cohort (**Table S1**). The results revealed that CMTM6 was upregulated in six [breast invasive carcinoma (BLCA), colon adenocarcinoma (COAD), esophageal carcinoma (ESCA), kidney renal papillary cell carcinoma (KIRP), stomach adenocarcinoma (STAD), and thyroid carcinoma (THCA)] and downregulated in six [cholangiocarcinoma (CHOL), kidney renal clear cell carcinoma (KIRC), liver hepatocellular carcinoma (LIHC), lung adenocarcinoma (LUAD), lung squamous cell carcinoma (LUSC), and pheochromocytoma and paraganglioma (PCPG)] cancer types relative to that in normal tissues (**Figure 1A**).

Additionally, we investigated CMTM6 protein expression from the HPA cohort, which presented CMTM6 protein expression in 14 different tumor types. High or medium CMTM6 expression levels were observed in pancreatic adenocarcinoma (PAAD) (75%), LIHC (66.7%), BLCA (63.6%), STAD (63.6%), ovarian serous cystadenocarcinoma (OV) (54.5%), uterine corpus endometrial carcinoma (UCEC) (33.3%), head and neck squamous cell carcinoma (HNSC) (25%), prostate adenocarcinoma (PRAD) (25%), lymphoma (LYMP) (25%), THCA (25%), testicular germ cell tumors (TGCT) (18.2%), breast invasive carcinoma (BRCA) (11.1%), cervical squamous cell carcinoma and endocervical adenocarcinoma (CESC) (10%), and COAD (8.3%) (**Figures 1B, C**). These results indicated CMTM6 may play different roles in cancer progression.

### Frequencies of CMTM6 Mutations and Copy Number Alterations in Multiple Cancers

**Figure 2:**
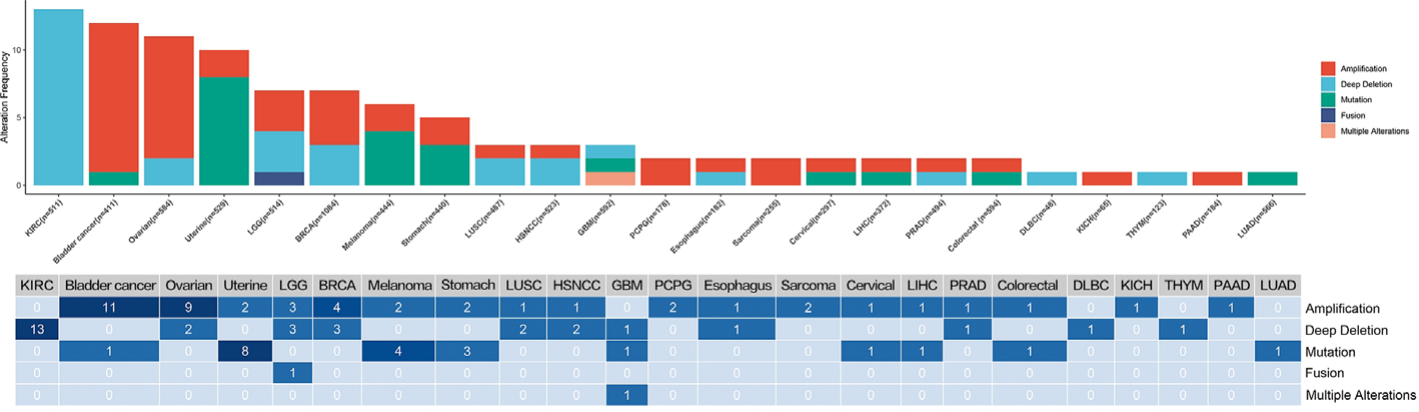
The frequency of CMTM6 copy number alterations and mutations in multiple cancers.

CMTM6 mutations and copy number alterations were investigated using cBioPortal. The results indicated low CMTM6 mutation frequencies in multiple cancer types, with the highest CMTM6 mutation frequency in uterine cancer. Amplification accounted for 0.49% (46/9,477) of the copy number alterations, while insertions and deletions (indels) comprised 0.32% (30/9,477) (**Figure 2**).

### Association Between *CMTM6* mRNA Expression and PD-L1 Protein Expression

**Figure 3:**
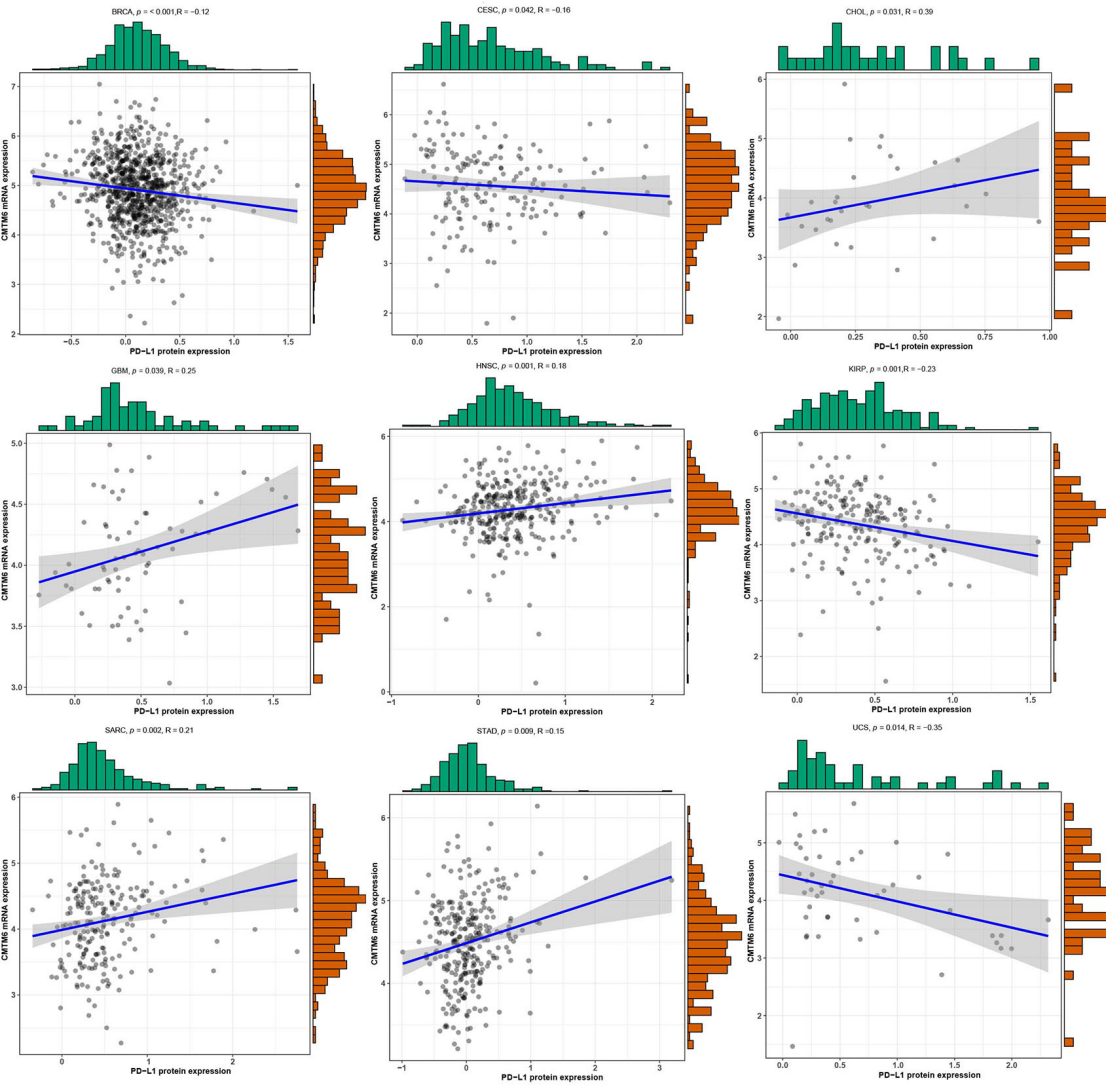
The correlation of CMTM6 expression with PD-L1 protein expression. High CMTM6 expression was positively associated with PD-L1 protein expression in cholangiocarcinoma (CHOL), head and neck squamous cell carcinoma (HNSC), sarcoma (SARC), stomach adenocarcinoma (STAD), whereas, negative correlation was observed in breast invasive carcinoma (BRCA), cervical squamous cell carcinoma and endocervical adenocarcinoma (CESC), kidney renal papillary cell carcinoma (KIRP), and uterine carcinosarcoma (UCS).

We investigated the correlation between expression of *CMTM6* mRNA and PD-L1 protein expression using PD-L1 data from the TCPA cohort (**Table S2**). Our results revealed that *CMTM6* mRNA expression was associated with PD-L1 protein expression in BRCA, CESC, CHOL, glioblastoma multiforme (GBM), HNSC, KIRP, sarcoma (SARC), STAD, and uterine carcinosarcoma (UCS) (**Figure 3**, **Figure S1**).

### Correlation Between CMTM6 Expression, Tumor Mutational Burden, and Microsatellite Instability

**Figure 4:**
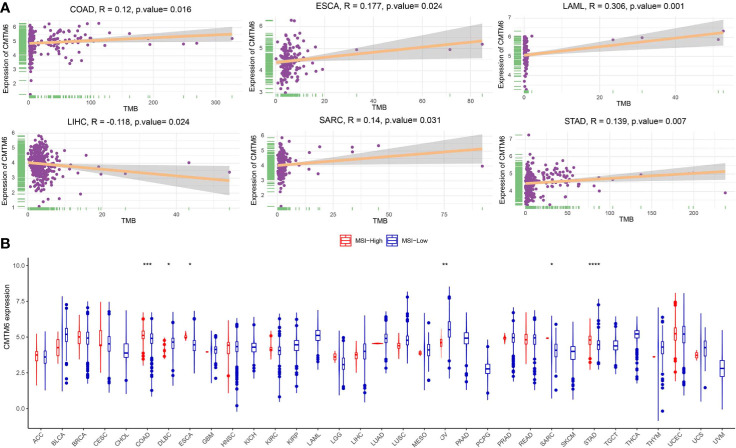
Correlation of CMTM6 expression with tumor mutational burden (TMB) and microsatellite high instability (MSI-H). **(A)** CMTM6 expression was associated with TMB in colon adenocarcinoma (COAD), esophageal carcinoma (ESCA), acute myeloid leukemia (LAML), liver hepatocellular carcinoma (LIHC), sarcoma (SARC), and stomach adenocarcinoma (STAD). **(B)** CMTM6 expression was associated with MSI-H in colon adenocarcinoma (COAD), lymphoid neoplasm diffuse large B-cell lymphoma (DLBC), esophageal carcinoma (ESCA), ovarian serous cystadenocarcinoma (OV), sarcoma (SARC), and stomach adenocarcinoma (STAD). *p < 0.05; **p < 0.01, ***p < 0.001, ****p < 0.0001.

TMB and MSI have been associated with cancer immunotherapeutic response and prognosis. In this study, we assessed TMB across 33 cancer types using the MuTect2 pipeline and found that TMB was the highest for skin cutaneous melanoma (**Figure S2**). We further evaluated the relationship between CMTM6 expression and TMB and showed that CMTM6 expression was correlated with TMB in COAD, ESCA, acute myeloid leukemia (LAML), LIHC, SARC, and STAD (**Figure 4A**), while no relationship was observed in the other 27 cancers (**Figure S3**). Further, we evaluated the association between CMTM6 expression and MSI status in different tumors. Our results indicated that MSI-H occurred the most frequently in UCEC, lymphoid neoplasm diffuse large B-cell lymphoma (DLBC), and COAD (**Figure S4**), and CMTM6 expression was positively associated with MSI-H in COAD, ESCA, SARC, and STAD. However, CMTM6 expression was negatively correlated with MSI-H in DLBC and OV (**Figure 4B**).

### Relationship Between CMTM6 Expression and Tumor Immune Microenvironment

**Figure 5:**
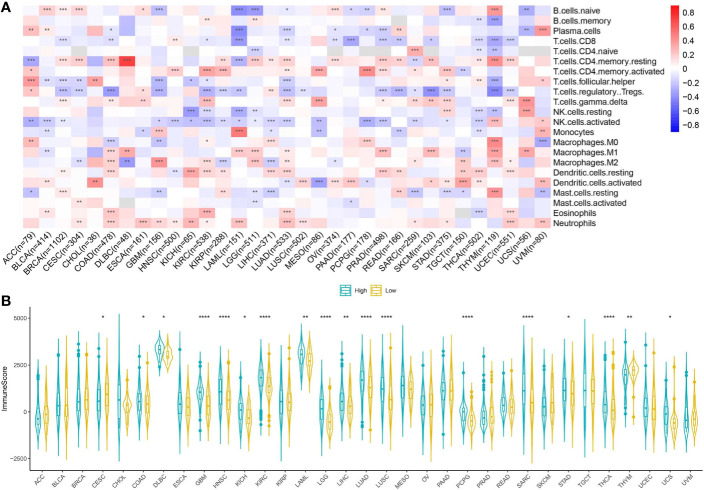
Relationship between CMTM6 expression and tumor microenvironment factors. **(A)** Correlation of CMTM6 expression with immune infiltrate subtypes across 33 cancer types. Red color represents positive correlation and blue color represents negative correlation. **(B)** Correlation of CMTM6 expression and immune scores of 33 different cancer types. *p < 0.05; **p < 0.01, ***p < 0.001, ****p < 0.0001.

We investigated the relationship between CMTM6 expression and immune cell infiltrates in the tumor microenvironment using CIBERSORT. The correlation between CMTM6 expression and tumor-infiltrating immune cells differed for different cancers. Interestingly, we found that high CMTM6 expression was positively associated with neutrophil infiltration in 14 cancer types (**Figure 5A**).

We applied the ESTIMATE algorithm to calculate the immune score for each sample in the TCGA cohort. To explore the potential correlation between CMTM6 expression and immune scores, patients were divided into high and low CMTM6 expression groups using the median CMTM6 expression as the cutoff value. CMTM6 expression was positively related to immune score in COAD, DLBC, GBM, HNSC, kidney chromophobe (KICH), KIRC, LAML, brain lower grade glioma (LGG), LIHC, LUAD, LUSC, PCPG, SARC, STAD, THCA, and UCS (**Figure 5B**). The results suggested that CMTM6 expression was associated with high immune infiltration in some cancer types.

### Correlations Between *CMTM6* Expression and Immune Checkpoint-Associated Genes

**Figure 6:**
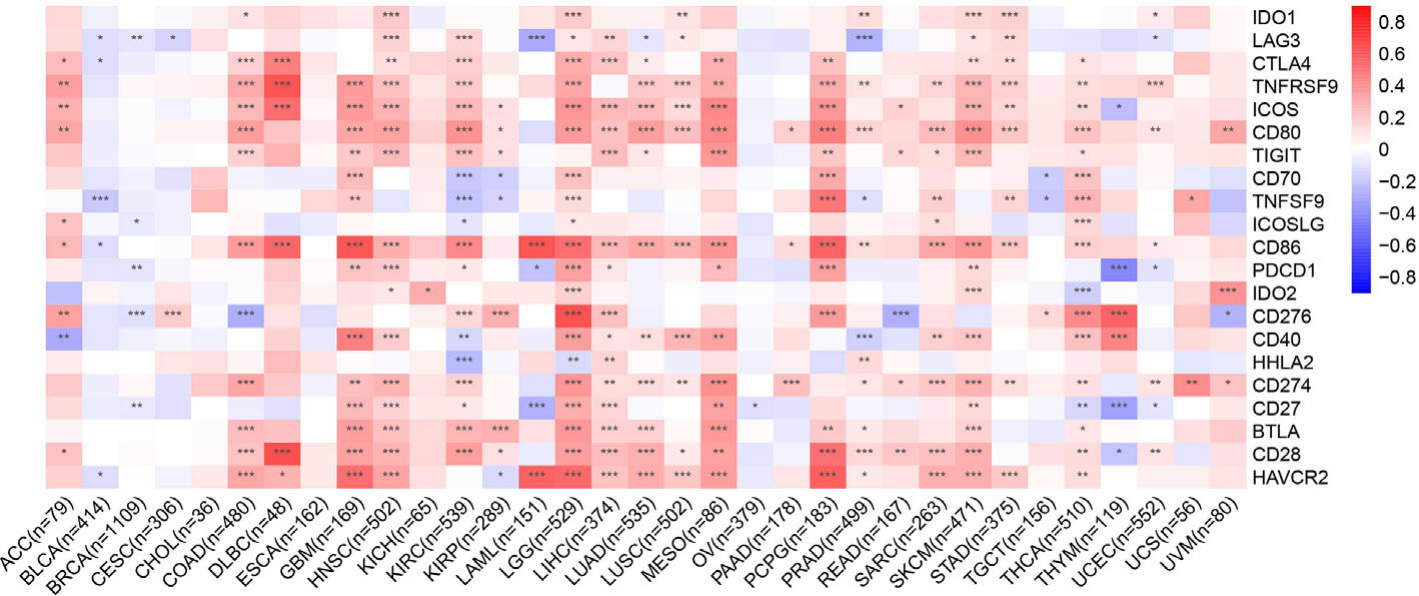
Heatmap representation of the correlation between CMTM6 expression and checkpoint-associated genes across 33 cancer types. Red color represents positive correlation and blue color represents negative correlation. *p < 0.05; **p < 0.01; ***p < 0.001.

Immune checkpoint-associated genes play an important role in immune escape (16). We further explored correlations between *CMTM6* expression and immune checkpoint-associated genes, including *IDO1*, *LAG3*, *CTLA4*, *TNFRSF9*, *ICOS*, *CD80*, *TIGIT*, *CD70*, *TNFSF9*, *ICOSLG*, *CD86*, *PDCD1*, *IDO2*, *CD276*, *CD40*, *HHLA2*, *CD274*, *CD27*, *BTLA*, *CD28*, and *HAVCR2* across 33 types of cancer from the TCGA cohort. We found that *CMTM6* expression was closely related to almost all immune checkpoint-associated genes except for *TIGIT* in LGG (**Figure 6**). Furthermore, *CMTM6* expression was not associated with most immune checkpoint-associated genes in CESC, CHOL, ESCA, KICH, OV, and UCS.

### CMTM6 Is a Prognostic Biomarker in Multiple Cancers

**Figure 7:**
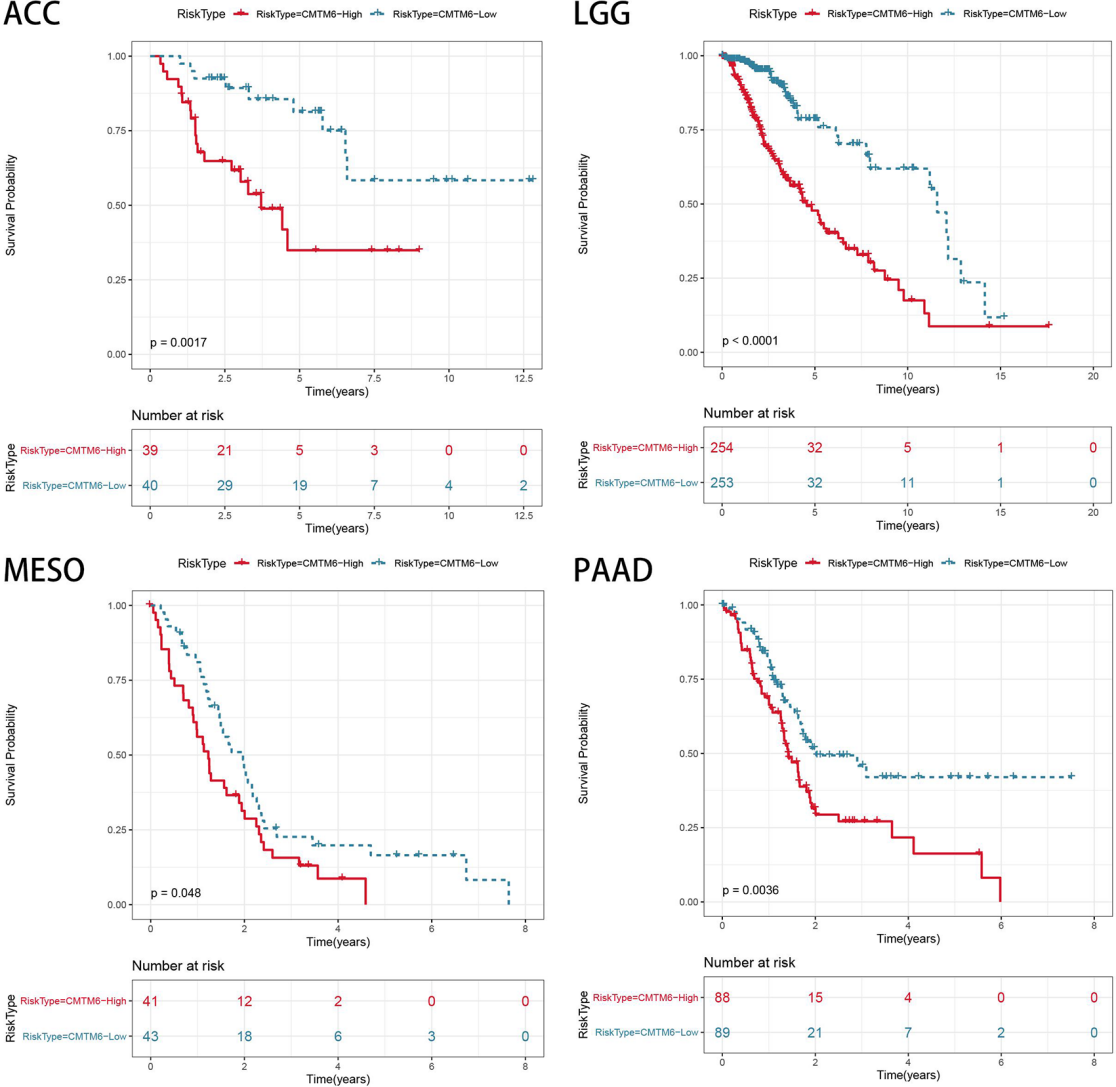
Association of CMTM6 expression with patient overall survival in pan-cancer. High expression of CMTM6 predicts poor overall survival (OS) of adrenocortical carcinoma (ACC), lower grade glioma (LGG), mesothelioma (MESO), and pancreatic adenocarcinoma (PAAD). Red color represents high CMTM6 expression and blue color represents low CMTM6 expression.

The relationship between CMTM6 expression and patient OS was analyzed using the TCGA cohort. High CMTM6 expression was an unfavorable factor for patient OS in adrenocortical carcinoma (ACC) (p = 0.0023), LGG (p < 0.001), mesothelioma (MESO) (p = 0.048), and PAAD (p = 0.0036) (**Figure 7**). However, high CMTM6 expression was not associated with prognosis in 29 other cancer types (**Figure S5**). These results suggested that CMTM6 expression may play a promoter role in ACC, LGG, MESO, and PAAD tumors.

## Discussion

Immune checkpoint inhibitor therapy has emerged as a critical treatment option in multiple cancer types (17). However, it is effective in a minority of patients; only 12.6% of all cancer patients benefit from immune checkpoint inhibitors (18). Therefore, exploration of predictive biomarkers for successful treatment or combined strategies is warranted to increase the therapeutic response rate to immune checkpoint inhibitors. Recently, CMTM6 expression was shown to be correlated with an improved response to PD-1 inhibitors (19). Given the crucial role of CMTM6 in tumor-related immune responses, we investigated the association between pan-cancer CMTM6 expression patterns and tumor immune microenvironments. In the present study, we analyzed CMTM6 mRNA and protein expression in multiple cancers using TCGA and HPA cohorts, respectively. We found high heterogeneity in the levels of *CMTM6* expression in different cancer types.

Previous studies have shown that PD-L1 protein expression is positively associated with response to anti-PD-1 immunotherapy (20–22). Therefore, fully understanding the regulation mechanism of PD-L1 protein expression is required to improve the efficacy of PD-1/PD-L1 inhibitors. Post-translational regulation is an important mechanism for regulating PD-L1 expression (23). Two recent studies have confirmed that CMTM6 stabilizes PD-L1 protein expression to attenuate T-cell immune surveillance (7, 8). We, thus, evaluated the correlation between CMTM6 expression and PD-L1 protein expression. PD-L1 expression was positively correlated with CMTM6 expression in CHOL, GBM, HNSC, SARC, and STAD, implying that high CMTM6 expression could respond favorably to anti-PD-1/PD-L1 immunotherapy in these tumor types.

Increasing evidence supports TMB as a potential biomarker of immune checkpoint inhibitor response in most cancers (24–26). These studies suggested that a higher burden of non-synonymous mutations in tumors facilitated the increased formation of neoantigens, making the tumor more immunogenic and, thus, improving the clinical response to immunotherapy. In this study, we evaluated the association between CMTM6 and TMB, revealing that CMTM6 expression was not associated with TMB in most cancer types, except in COAD, ESCA, LAML, LIHC, SARC, and STAD. We found that these associations were usually related to cancer type. MSI is caused by the insertion or loss of base pairs in the microsatellite region owing to replication errors. Recent studies have also shown that MSI and/or mismatch-repair deficiency (dMMR) could serve as potential biomarkers and predict the efficacy of immunotherapy, irrespective of cancer type (27). In patients with advanced dMMR or MSI-H cancers treated with pembrolizumab, Le et al. reported that objective response was observed in 53% of patients and 64% of patients experienced 2-year survival (28). The targeted monoclonal antibody nivolumab has also demonstrated effectiveness in dMMR or MSI-H colorectal cancer (29). Based on the importance of microsatellite instability in tumor immunotherapy, we evaluated the MSI status of tumor patients in the TCGA cohort and further analyzed the correlation between MSI-H and CMTM6 expression. We found that CMTM6 expression was positively related to MSI-H in UCEC, DLBC, and COAD. It is worth emphasizing that high CMTM6 expression in these tumors could identify patients who might respond favorably to PD-1/PD-L1 antibody immunotherapy.

Tumor immune infiltrating cells migrate from blood to tumor tissues and play an important role in immune regulation. Increasing numbers of studies have shown that tumor immune infiltrating cells are closely related to the efficacy of immune checkpoint inhibition and prognosis (30–32). To elucidate the relationship between CMTM6 expression and diverse infiltrating lymphocytes, we used CIBERSORT to examine the relative fractions of infiltrating immune cell types across 33 cancer types. We found that these associations depended on tumor type. CMTM6 expression was associated with invasive neutrophils in most tumors. Tumor-associated neutrophils are generally considered to be tumor-promoting agents in many tumor types (33). We speculate that CMTM6 expression may play a role in regulating tumor cells by inducing neutrophil infiltration. Further *in vitro* and *in vivo* research is warranted to validate the relationship between CMTM6 expression and neutrophils. A recent study showed that CMTM6 expression was positively associated with M2-like macrophage infiltration in oral squamous cell carcinoma (34). Furthermore, we evaluated the immune score of patients with tumor from the TCGA cohort using the ESTIMATE algorithm and found that high CMTM6 expression was associated with higher immune infiltration score in most tumors. This further demonstrated that changes in CMTM6 expression can affect immune cell infiltration in the tumor microenvironment. However, *ex vivo* and *in vivo* studies are required to further support the findings of our study. Recent studies have shown that CMTM6 plays an oncogenic role and is associated with poor prognosis in gliomas, hepatocellular carcinoma, and LUAD (2, 35, 36). However, there is limited information regarding the prognostic value of CMTM6 in other solid cancer types. Our results indicated that high CMTM6 expression was associated with poor clinical prognosis in ACC, GBM, LAML, LIHC, MESO, SARC, THYM, and UVM and that CMTM6 may play a promoting role in tumor progression.

## Conclusions

In conclusion, we herein report that CMTM6 is heterogeneously expressed in diverse cancers and its expression is correlated with the tumor immune microenvironment and pan-cancer prognosis. High CMTM6 expression was associated with expression of immune checkpoint-associated genes and poor prognosis in diverse prevailing cancers.

This manuscript has been released as a pre-print at ResearchSquare (Yan Wang et al.) (37).

## Data Availability Statement

The datasets presented in this study can be found in online repositories. The names of the repository/repositories and accession number(s) can be found in the article/**Supplementary Material**.

## Author Contributions

YW conceived, designed, planned the study, and interpreted the results. MZ, YZ, and HP analyzed the data. SG and SZ acquired the data. MZ and YZ drafted the manuscript. All authors revised and reviewed this work, and gave their final approval of the submitted manuscript. All authors contributed to the article and approved the submitted version.

## Funding

This study was supported by the Natural Science Foundation of China (Grant No. 81673024), the Natural Science Foundation of Heilongjiang Province (Grant No. LH2019H040, JJ2018LX0182), the N10 Program of Harbin Medical University Cancer Hospital (Grant No. nN10PY2017-04), and the Haiyan Science Fund of Harbin Medical University Cancer Hospital (Grant No. JJZD2017-06).

## Conflict of Interest

The authors declare that the research was conducted in the absence of any commercial or financial relationships that could be construed as a potential conflict of interest.

## Abbreviations

ACC, adrenocortical carcinoma; BLCA, bladder urothelial carcinoma; BRCA, breast invasive carcinoma; CESC, cervical squamous cell carcinoma and endocervical adenocarcinoma; CHOL, cholangiocarcinoma; CMTM6, CKLF-like MARVEL transmembrane domain-containing 6; COAD, colon adenocarcinoma; DLBC, lymphoid neoplasm diffuse large B-cell lymphoma; ESCA, esophageal carcinoma; GBM, glioblastoma multiforme; HNSC, head and neck squamous cell carcinoma; HPA, human protein atlas; KICH, kidney chromophobe; KIRC, kidney renal clear cell carcinoma; KIRP, kidney renal papillary cell carcinoma; LAML, acute myeloid leukemia; LGG, brain lower grade glioma; LIHC, liver hepatocellular carcinoma; LUAD, lung adenocarcinoma; LUSC, lung squamous cell carcinoma; MESO, mesothelioma; MSI, microsatellite instability; MSI-H, microsatellite high instability; MSS, microsatellite stable; PCPG, pheochromocytoma and paraganglioma; PD-1, programmed death-1; PD-L1, programmed death-ligand 1; SARC, sarcoma; STAD, stomach adenocarcinoma; TCGA, The Cancer Genome Atlas; TCPA, The Cancer Proteome Atlas; THCA, thyroid carcinoma; THYM, thymoma; UCS, uterine carcinosarcoma; UV, uveal melanoma
