# Supplementary material for: Prognostic Implications of Pan-Cancer CMTM6 Expression and Its Relationship with the Immune Microenvironment
Source: Front Oncol. 2021 Jan 20;10:585961. doi: 10.3389/fonc.2020.585961 (PMC7855963; doi:10.3389/fonc.2020.585961)
Supplement: Supplementary file 1 [file DataSheet_1.pdf]

Table-S1 Overview of TCGA datasets used for the analysis.

Cancer Type	Full Name	Cancer Samples	Normal Smaples	Total Smaples
ACC	Adrenocortical Carcinoma	79	0	79
BLCA	Bladder Urothelial Carcinoma	414	19	433
BRCA	Breast Invasive Carcinoma	1109	113	1222
CESC	Cervical Squamous Cell Carcinoma and Endocervical Adenocarcinoma	306	3	309
CHOL	Cholangiocarcinoma	36	9	45
COAD	Colon Adenocarcinoma	480	41	521
DLBC	Lymphoid Neoplasm Diffuse Large B-cell Lymphoma	48	0	48
ESCA	Esophageal Carcinoma	162	11	173
GBM	Glioblastoma Multiforme	169	5	174
HNSC	Head and Neck Squamous Cell Carcinoma	502	44	546
KICH	Kidney Chromophobe	65	24	89
KIRC	Kidney Renal Clear Cell Carcinoma	539	72	611
KIRP	Kidney Renal Papillary Cell Carcinoma	289	32	321
LAML	Acute Myeloid Leukemia	151	0	151
LGG	Brain Lower Grade Glioma	529	0	529
LIHC	Liver Hepatocellular Carcinoma	374	50	424
LUAD	Lung Adenocarcinoma	535	59	594
LUSC	Lung Squamous Cell Carcinoma	502	49	551
MESO	Mesothelioma	86	0	86
OV	Ovarian Serous Cystadenocarcinoma	379	0	379
PAAD	Pancreatic Adenocarcinoma	178	4	182
PCPG	Pheochromocytoma and Paraganglioma	183	3	186
PRAD	Prostate Adenocarcinoma	499	52	551
READ	Rectum Adenocarcinoma	167	10	177
SARC	Sarcoma	263	2	265
SKCM	Skin Cutaneous Melanoma	471	1	472
STAD	Stomach Adenocarcinoma	375	32	407
TGCT	Testicular Germ Cell Tumors	156	0	156
THCA	Thyroid Carcinoma	510	58	568
THYM	Thymoma	119	2	121
UCEC	Uterine Corpus Endometrial Carcinoma	552	35	587
UCS	Uterine Carcinosarcoma	56	0	56
UVM	Uveal Melanoma	80	0	80
Total		10363	730	11093

Table-S2 Overview of TCPA datasets used for analysis of PD-L1 protein expression

Cancer Type	Full Name	Cancer Samples
ACC	Adrenocortical Carcinoma	46
BLCA	Bladder Urothelial Carcinoma	340
BRCA	Breast Invasive Carcinoma	867
CESC	Cervical Squamous Cell Carcinoma and Endocervical Adenocarcinoma	169
CHOL	Cholangiocarcinoma	30
COAD	Colon Adenocarcinoma	352
DLBC	Lymphoid Neoplasm Diffuse Large B-cell Lymphoma	33
ESCA	Esophageal Carcinoma	105
GBM	Glioblastoma Multiforme	67
HNSC	Head and Neck Squamous Cell Carcinoma	331
KICH	Kidney Chromophobe	63
KIRC	Kidney Renal Clear Cell Carcinoma	439
KIRP	Kidney Renal Papillary Cell Carcinoma	205
LGG	Brain Lower Grade Glioma	423
LIHC	Liver Hepatocellular Carcinoma	181
LUAD	Lung Adenocarcinoma	357
LUSC	Lung Squamous Cell Carcinoma	322
MESO	Mesothelioma	60
OV	Ovarian Serous Cystadenocarcinoma	258
PAAD	Pancreatic Adenocarcinoma	97
PCPG	Pheochromocytoma and Paraganglioma	78
PRAD	Prostate Adenocarcinoma	348
READ	Rectum Adenocarcinoma	127
SARC	Sarcoma	219
SKCM	Skin Cutaneous Melanoma	91
STAD	Stomach Adenocarcinoma	301
TGCT	Testicular Germ Cell Tumors	118
THCA	Thyroid Carcinoma	368
THYM	Thymoma	86
UCEC	Uterine Corpus Endometrial Carcinoma	403
UCS	Uterine Carcinosarcoma	48
UVM	Uveal Melanoma	12
Total		6944

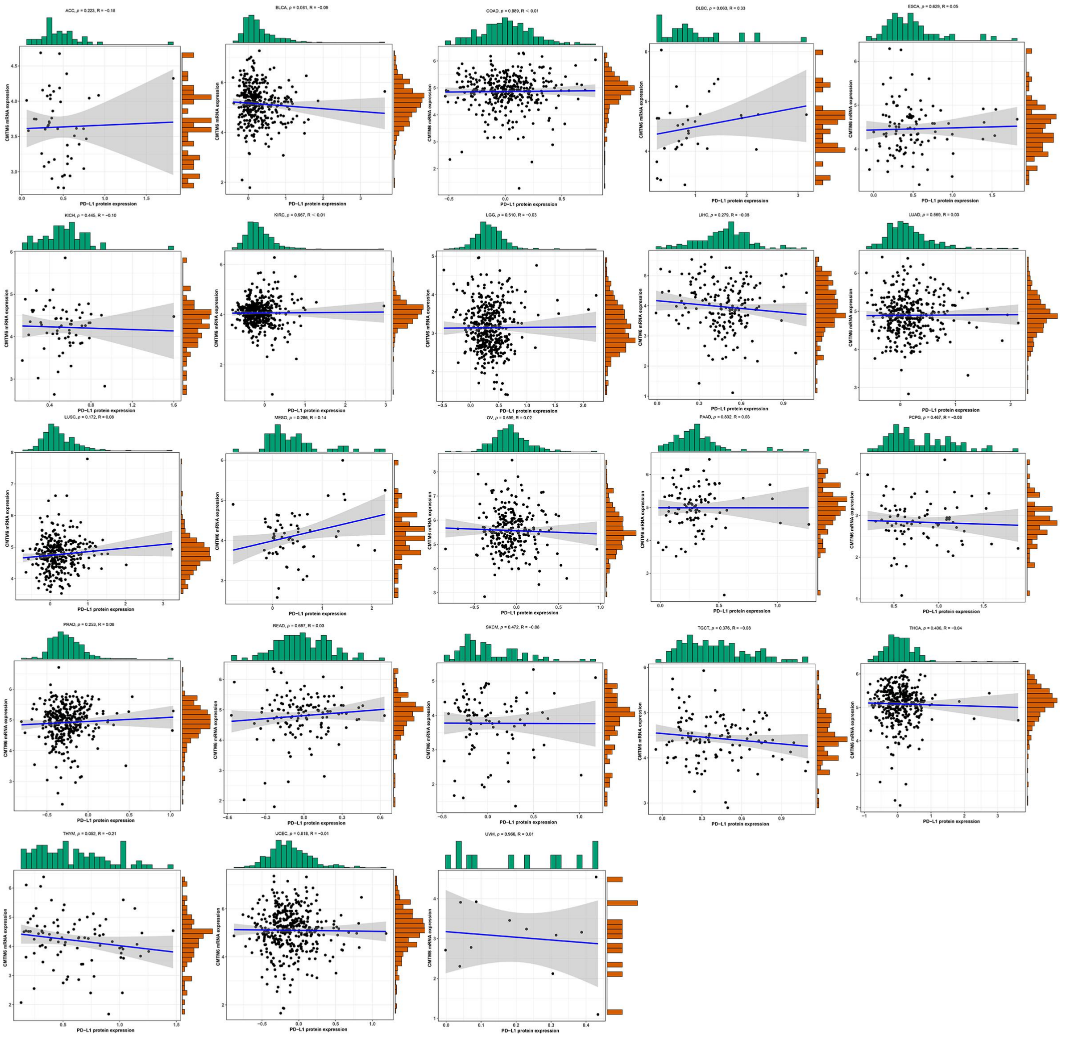

Fig. S1 Association between CMTM6 expression and PD-L1 protein expression. CMTM6 expression was not associated with ACC, BLCA, COAD, DLBC, ESCA, KICH, KIRC, LGG, LIHC, LUAD, LUSC, MESO, OV, PAAD, PCPG,PRAD, READ, SKCM, TGCT, THCA, THYM, UCEC, UVM.

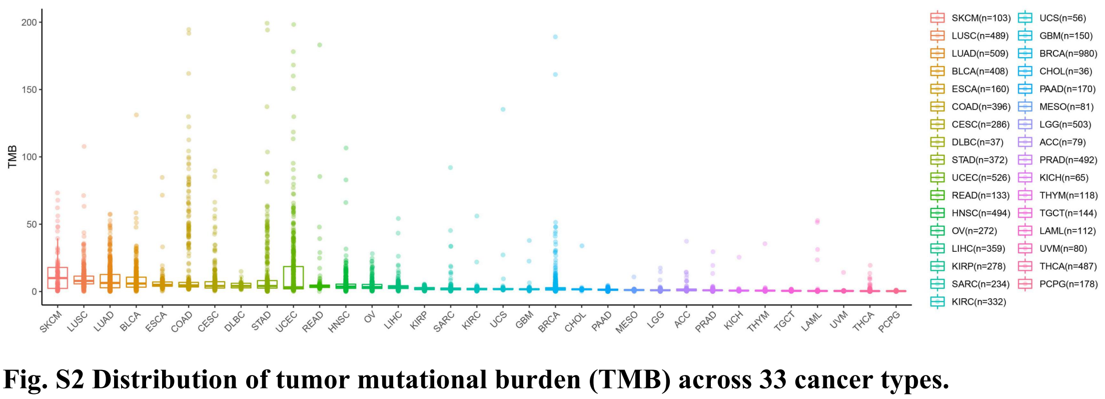

Fig. S2 Distribution of tumor mutational burden (TMB) across 33 cancer types.

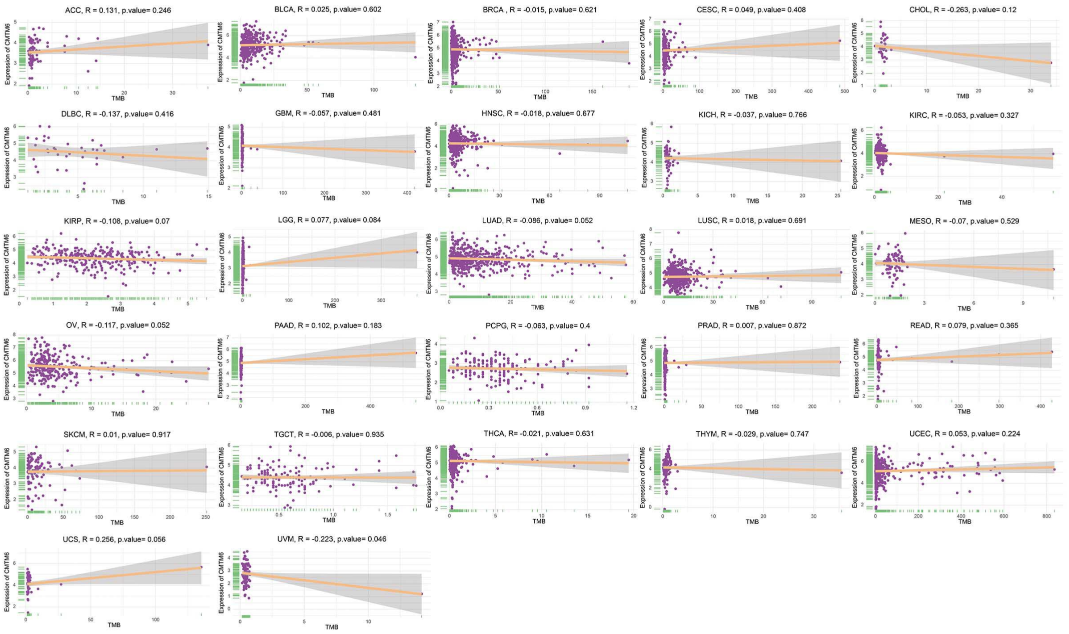

Fig. S3 Association between CMTM6 expression and tumor mutational burden (TMB). CMTM6 expression was not associated with ACC, BLCA, BRCA, CESC, CHOL, DLBC, GBM, HNSC, KICH, KIRC, KIRP, LGG, LUAD, LUSC, MESO, OV, PAAD, PCPG, PRAD, READ, SKCM, TGCT, THCA, THYM, UCEC, UCS, and UVM.

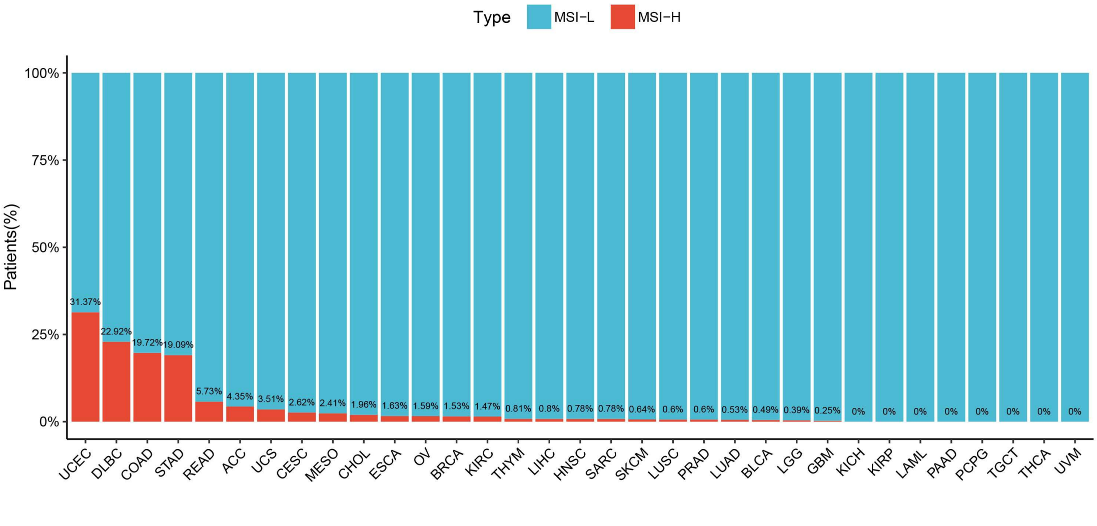

Fig. S4 Prevalence of microsatellite instability (MSI) across 33 cancer types.

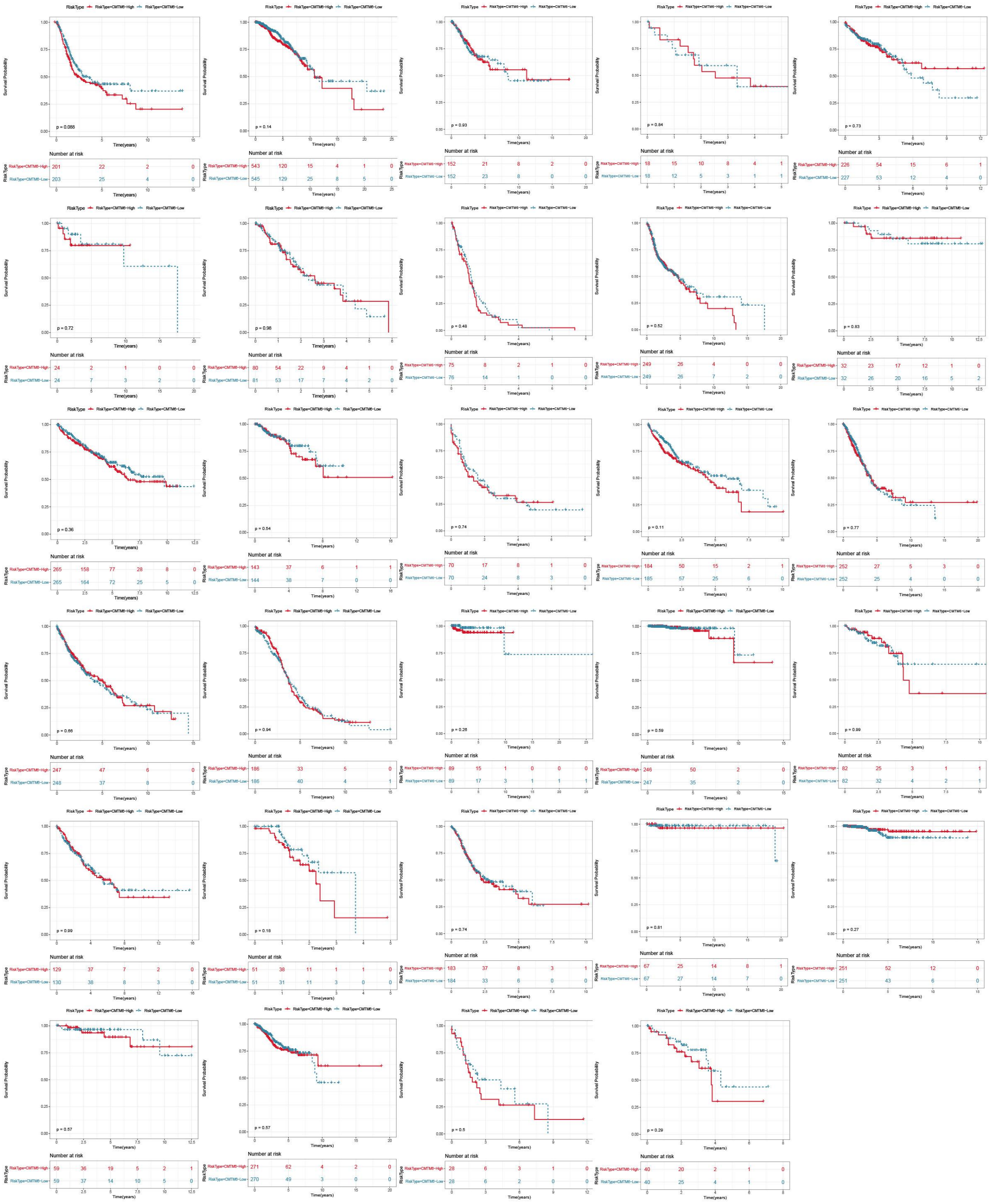

Fig. S5 Prognostic values of CMTM6 in 33 cancer types analyzed in the TCGA cohort. High expression of CMTM6 was not associated with overall survival in BLCA, BRCA, CESC, CHOL, COAD, DLBC, ESCA, GBM, HNSC, KICH, KIRC, KIRP, LAML, LIHC, LUAD, LUSC, OV, PCPG, PRAD, READ, SARC, SKCM, STAD, TGCT, THCA, THYM, UCEC, UCS and UVM.
